# Transferrin-targeted porous silicon nanoparticles reduce glioblastoma cell migration across tight extracellular space

**DOI:** 10.1038/s41598-020-59146-5

**Published:** 2020-02-11

**Authors:** Sana Sheykhzadeh, Meihua Luo, Bo Peng, Jacinta White, Youssef Abdalla, Tweety Tang, Ermei Mäkilä, Nicolas H. Voelcker, Wing Yin Tong

**Affiliations:** 1 Department of Pharmaceutical and Biological Chemistry, UCL School of Pharmacy, University College London, Brunswick Square, London, United Kingdom; 2 Drug Delivery, Disposition and Dynamics, Monash Institute of Pharmaceutics Science, Monash University, Parkville, Victoria, Australia; 3 Department of Biomedical Engineering, The Chinese University of Hong Kong, Shatin, New Territories Hong Kong; 4 Commonwealth Scientific and Industrial Research Organization (CSIRO), Clayton, Victoria, Australia; 5 Melbourne Centre for Nanofabrication, Victorian Node of the Australian National Fabrication Facility, Clayton, Victoria, Australia; 6 Department of Chemistry, City University of Hong Kong, Kowloon, Hong Kong; 7 Industrial Physics Laboratory, Department of Physics and Astronomy, University of Turku, Turku, Finland; 8 Department of Materials Science and Engineering, Monash University, Clayton, Victoria, Australia

**Keywords:** Biomaterials, CNS cancer

## Abstract

Mortality of glioblastoma multiforme (GBM) has not improved over the last two decades despite medical breakthroughs in the treatment of other types of cancers. Nanoparticles hold tremendous promise to overcome the pharmacokinetic challenges and off-target adverse effects. However, an inhibitory effect of nanoparticles by themselves on metastasis has not been explored. In this study, we developed transferrin-conjugated porous silicon nanoparticles (Tf@pSiNP) and studied their effect on inhibiting GBM migration by means of a microfluidic-based migration chip. This platform, designed to mimic the tight extracellular migration tracts in brain parenchyma, allowed high-content time-resolved imaging of cell migration. Tf@pSiNP were colloidally stable, biocompatible, and their uptake into GBM cells was enhanced by receptor-mediated internalisation. The migration of Tf@pSiNP-exposed cells across the confined microchannels was suppressed, but unconfined migration was unaffected. The pSiNP-induced destabilisation of focal adhesions at the leading front may partially explain the migration inhibition. More corroborating evidence suggests that pSiNP uptake reduced the plasticity of GBM cells in reducing cell volume, an effect that proved crucial in facilitating migration across the tight confined tracts. We believe that the inhibitory effect of Tf@pSiNP on cell migration, together with the drug-delivery capability of pSiNP, could potentially offer a disruptive strategy to treat GBM.

## Introduction

Glioblastoma multiforme (GBM) is the most prevalent and biologically aggressive type of primary brain tumour in adults^1^. Standard treatment is maximal surgical resection of a tumour followed by radiotherapy and temozolomide as an adjuvant chemotherapy^2^. Despite these advanced treatments, the survival rate of GBM patients is still less than 5% over five years, with a median overall survival of merely 15–23 months^3^. The factors that contribute to the high mortality are multifactorial. First and foremost, the diffuse invasion of GBM into brain parenchyma precludes complete surgical resection which leads to high recurrence^4^. Recurring GBM are usually multi-drug resistant, rendering chemotherapy ineffective^5^. On rare occasions, the high invasion and migration potential even leads to extracranial metastases^6^.

The mechanisms of GBM invasion and migration are complex and encompasses the regulation of tumour microenvironment and of the molecular arrangement within the migrating GBM cells^7^. To enable migration across the small perivascular space, GBM cells have been shown to reduce their volume by releasing cytoplasmic fluid^8^. The reduction in cell size is particularly instrumental to GBM invasion into healthy brain tissue, since they migrate along pre-existing structures with high mechanical rigidity like blood vessels and myelinated nerve fibres^9^. Besides the dramatic change in cell volume, GBM cells also rearrange the turnover of focal adhesions (FA) to facilitate migration. FA are temporal self-assembling complexes, which help the cells to form anchors with the extracellular matrix (ECM). Dynamics of several FA proteins, including integrin, vinculin, talin, and α-actinin, were shown to correlate to GBM cell traction force generation, which is associated with their adhesion to ECM and migration^10^.

Deriving therapeutic counter measures against aforementioned infiltrative migration mechanisms employed by a cancer cell (such as the use of small molecules^11^, peptides^12^, RNA interference^13^) has been an obvious approach to intervene cancer progression^14^. Despite those discoveries, the pharmacokinetic challenges such as rapid degradation, clearance of those small molecules, and off-target adverse effects are still problematic in clinical translation. Their applications in GBM is particularly difficult, since the penetration of those agents across the blood–brain barrier (BBB) and targeted delivery into GBM are still the major bottlenecks^15^.

Nanomedicines offer unprecedented advantages in targeted drug delivery into tumours, such as enhanced cellular uptake, controlled release of drugs, and protection of the agents from premature degradation^16^. A range of nanoparticles (NP) have been developed to deliver small molecular drugs to inhibit cancer cell migration^17–19^. Interestingly, a few studies reported that NP treatments alone are inhibitory to cancer cell invasion of liver^20^, cervical^21^, and breast^22^ origins. However, studies on the potential of NP treatments in attenuating GBM invasion and migration are absent, despite the hallmark of GBM being their infiltrative phenotype.

We sought to systematically investigate the effect of NP treatment on the migration potential of GBM cells. Porous silicon nanoparticle (pSiNP) were chosen as a model. pSiNP is a non-polymeric NP which possesses high surface area and biocompatibility. pSiNP also exhibits good biodegradability, the rate of which can be tuned over a wide time window (minutes to months) via surface modification to suit the desired biomedical applications^23^. The dissolution product is silicic acid the non-toxic bioavailable form of silicon in the body^24,25^. The versatile surface chemistry of pSiNP allows a wide range of conjugation chemistries^24^. We conjugated pSiNP with human transferrin (Tf) to target GBM cells. Tf receptor (TfR) is one of the most commonly exploited cell surface targets for GBM, and the over-expression of TfR is conserved across the GBM biopsies^26^. TfR expression is also reported at the vessel side of microvascular endothelial cells at the BBB, and the use of Tf as a targeting molecule has been reported to enhance shuffling therapeutics across BBB^27^.

Most of the *in vitro* studies on cancer cell migration quantify migration by means of the scrape migration assay, whereby the speed of cell patches closure is positively correlated to their motility^28^. However this assay has several limitations, such as the absence of chemotaxis-related directional migration^29^, and the absence of a tightly confined microenvironment, that mimics the characteristic perivascular space that GBM cells infiltrate^30^. Transwell models, which gauge cell migration through a perforated membrane with micron-sized pores, provide a better option, but the setup is largely incompatible to high-content imaging modalities and time-resolved studies that are essential to gaining mechanistic insights^31^. Microfluidic chips constructed from transparent polymers and coverslips are becoming a popular option for oncology studies as they allow the implementation of chemotaxis-driven migration and high-content imaging^32^.

In this work, we systematically studied the influence of Tf-modified pSiNP (Tf@pSiNP) on GBM migration in a microfluidic-based cell migration chip. The chip comprised of microchannels that resemble the micron-scale perivascular space in brain parenchyma. We showed that Tf@pSiNP enhanced internalisation into GBM U87 cells. Although Tf@pSiNP were highly biocompatible and did not significantly affect ATP production in cells, Tf@pSiNP treatment significantly discouraged U87 migration across the microchannels. We also observed that the extent of pSiNP uptake was negatively correlated to the success of cell migration across the microchannels. The potential mechanisms of the inhibition on GBM migration by pSiNP were further studied. Focal adhesions (FA) at the leading fronts of migrating U87 cells which had internalised the pSiNP appeared to be destabilised. This phenomenon may represent a lack of the traction needed for cell migration. In addition, we demonstrated that Tf@pSiNP-internalised U87 cells with internalised Tf@pSiNP were more resistive to hypertonic pressure-induced reduction of cell volume. Since GBM migration across microchannels required a dramatic reduction of cell volume, we posit that the presence of Tf@pSiNP may inhibit U87 migration by attenuating the regulation of cell volume. Such inhibition has not been observed in a conventional scrape migration model which did not require the regulation of cell volume. In conclusion, our study proposes that Tf@pSiNP treatment could potentially inhibit GBM cells from migrating. Together with the promise of pSiNP in targeted drug delivery, we believe that Tf@pSiNP treatment could potentially applied to reduce GBM recurrence. We also envisage that the migration chip developed here also enables further study in the fundamental biology of GBM cell migration.

## Results

### Characterisation of Tf@pSiNP

**Figure 1:**
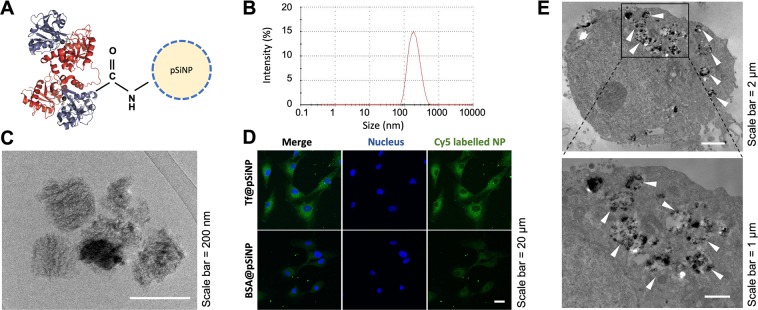
Characterisation of transferrin (Tf) modified porous silicon nanoparticles (Tf@pSiNP). (**A**) Schematic of Tf@pSiNP. Sizes of pSiNP and Tf are not in scale. (**B**) Hydrodynamic particle size distribution of Tf@pSiNP in PBS as measured by means of dynamic light scattering (DLS) using zeta-sizer. The zeta potential measured was −9.02 $\pm$ 0.64 mV. (**C**) Cryo-transmission electron microscopy image of Tf@pSiNP in PBS. (**D**) Cellular uptake of Tf@pSiNP and BSA@pSiNP in U87 cells imaged by laser scanning confocal microscopy. (**E**) Internalisation of Tf@pSiNP in U87 cells by cryo-TEM. The arrow heads show examples of Tf@pSiNP within the vesicles.

The conjugation of Tf onto pSiNP was performed as described in Fig. 1A, and the hydrodynamic particle size distribution and zeta potential were characterised by dynamic light scattering (DLS) with zeta-potential analyser. The shape of pSiNP was studied using cryogenic transmission electron microscopy (cryo-TEM) and Tf conjugation efficacy using inductively coupled plasma mass spectrometry (ICP-MS) respectively. DLS results, measured an average size of Tf@pSiNP of 182 ± 0.8 nm, and the particle size distribution was narrow as indicated by a polydispersity index of 0.1 (Fig. 1B). The dimensions of Tf@pSiNP revealed using cryo-TEM, corroborate the DLS results. The Tf@pSiNP were mostly plate shaped (Fig. 1C). ICP-MS results showed that 1 mg of Tf@pSiNP contains 0.61 ± 0.05 µg of Fe^2+^ ions. As the amount of Fe^2+^ per unit weight of Tf is known^33^, this translated to 0.38 ± 0.03 mg of Tf per 1 mg of pSiNP. The zeta potential of carboxylated pSiNP before reaction was −14.4 $\pm$ 1.5 mV, while Tf@pSiNP after reaction was −9.0 $\pm$ 0.6 mV. The unreacted carboxylate acid groups are believed to be the origin of the negative zeta potential.

To demonstrate the effect of Tf in enhancing pSiNP internalisation into GBM cells, bovine serum albumin-modified pSiNP (BSA@pSiNP) were prepared as a control to delineating the role of Tf functionalisation in biological activities. Under cryo-TEM, BSA@pSiNP appeared plate shaped (Supplementary Figure 1A) similar to Tf@pSiNP. Hydrodynamic particle size and zeta potential of BSA@pSiNP were 172 ± 4 nm and −9.9 ± 4.4, respectively (Supplementary Figure 1B), not significantly different from Tf@pSiNP (p = 0.10, and p > 0.99, respectively).

### Tf modification on pSiNP enhanced cellular uptake by GBM cells

Since Tf readily binds to TfR that is overexpressed in GBM cells, we aimed to evaluated whether the functionalisation of Tf would enhance Tf@pSiNP cellular uptake by GBM cells (U87). By imaging Cy5-labeled BSA@pSiNP and Tf@pSiNP in exposed cells using confocal microscopy, higher fluorescence intensity of Cy5-Tf@pSiNP (3 fold) was identified in the cytoplasm of GBM cells as compared to those exposed to Cy5-BSA@pSiNP (Fig. 1D). This result indicates that Tf modification enhanced the uptake of pSiNP into U87 cells. It should be noted that BSA@pSiNP was still taken up by U87 cells, although to a lesser degree. Unlike Tf, BSA is an inert protein abundantly present in serum and it is reported that BSA modification did not enhance NP uptake via clathrin-mediated receptor-mediated endocytosis^34^. This explains that the uptake was apparently slower as detailed in our previous work^35^. Additionally, the internalisation of Tf@pSiNP in U87 cells was confirmed by means of cryo-TEM imaging (Fig. 1E).

**Figure 2:**
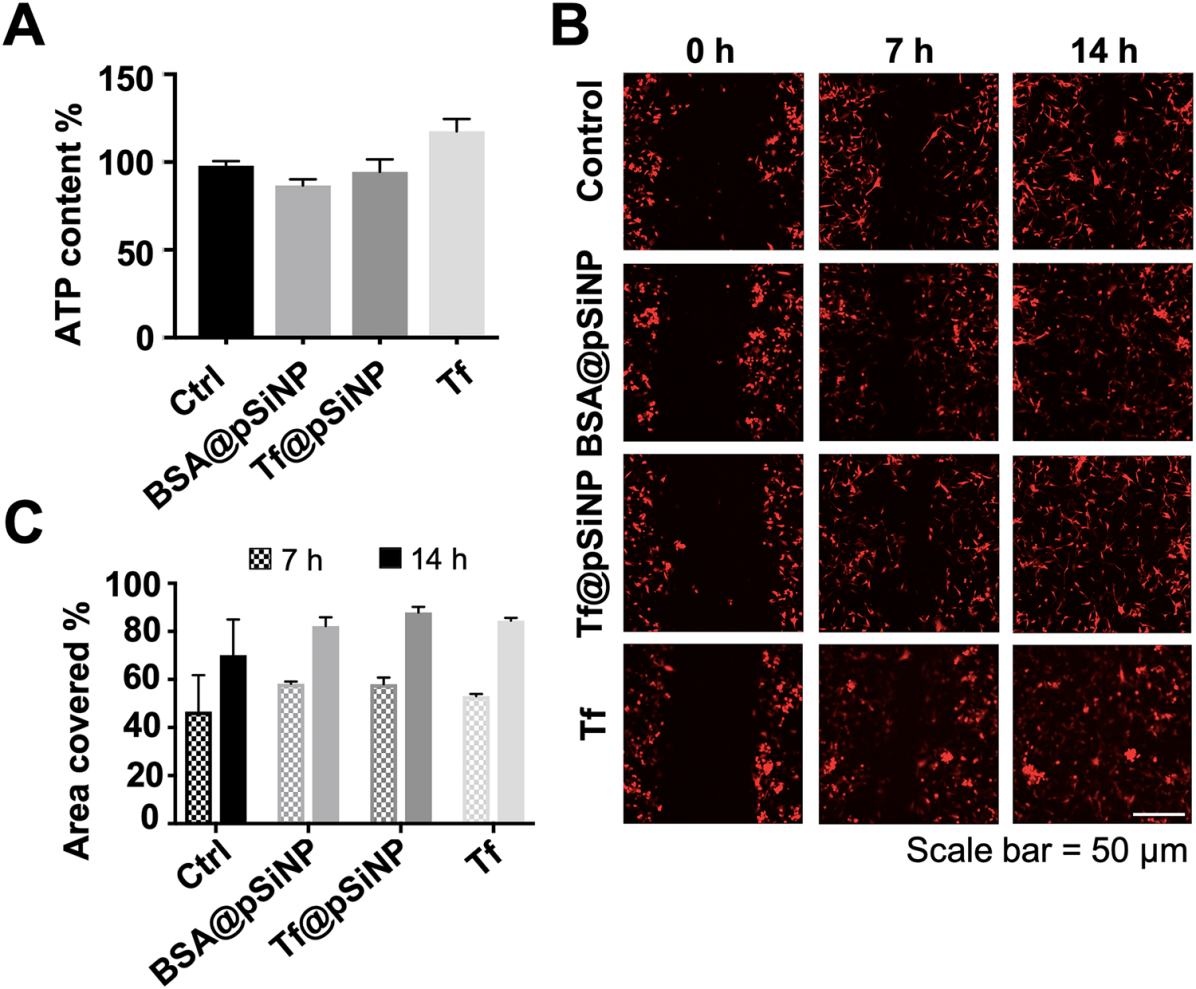
The effect of Tf@pSiNP treatment on GBM cells viability and migration (conventional scrape migration assay). (**A**) Normalised cell viability of U87 cells exposed to Tf-pSiNP for 8 h revealed via an ATP viability assay (n = 3). (**B**) Montage of U87 closing the scraped area in a conventional scrape migration model over the period of 14 h. Red indicates cytoplasmic mCherry representing U87 cell bodies. (**C**) Quantification of the cell coverage over the scraped area after 7 and 14 h. Experiments were triplicated (n = 3, error bar = ±1 SD. * Indicates p < 0.05. Untreated (ctrl), BSA-conjugated pSiNP treated (BSA@pSiNP), and free transferrin (Tf) treated cells were put as controls.

Prior to investigating the biological roles of Tf@pSiNP in GMB cells, biocompatibilities of Tf@pSiNP and BSA@pSiNP with U87 cells were examined via an ATP bioluminescence assay. Tf at a concentration of 38 µg/ml which was equivalent to the concentration of Tf for the Tf@pSiNP samples was used as controls. It was shown that the ATP contents in both BSA@pSiNP and Tf@pSiNP treated U87 cells did not exhibit significant difference from untreated cells (Fig. 2A). Interestingly, cells exposed to Tf had a higher level of ATP, which may due to the increased metabolic activity enhanced by Fe^2+^ uptake^36^. This result suggests that BSA@pSiNP, Tf@pSiNP and Tf did not negatively impact on the viability nor ATP level in U87 cells.

### Towards a microfluidic GBM cell migration chip

Having confirmed that Tf-modification enhanced pSiNP cellular uptake, and was nontoxic to cells, we examined their effect on GBM cells migration using a conventional scrape-migration assay, which is one of the most common *in vitro* models in the studies of anti and pro migration parameters of cancer cells^37^. Time-lapse imaging of U87 cell migration showed that the cells treated with Tf@pSiNP, BSA@pSiNP and Tf alone closed the scraped area at a similar rate and covered the empty spaces after 14 h (Fig. 2B). Cell coverage at the end of the study was quantified to indicate of the GBM cells motility. The data suggest that there was no significant difference between their rates in closing the scraped area, indicating the limited impact of Tf@pSiNP on GBM cells motility (Fig. 2C). Interestingly, despite Tf exposure elevated ATP content in U87 cells, their migration was not significantly different to control and pSiNP exposed cells. However, it is important to note that the scrape-migration assay is highly simplistic compared to brain cancer cells migration in the brain microenvironment. For instance, chemotaxis and the cell shape modulations during migration in confined brain parenchyma were not considered here.

To better mimic the GBM migration characteristics, a “migration chip” model was used. The migration chip is composed of two main channels (500 µm wide) interconnected with 80 µm long microchannels with a cross-section of 3 µm (wide) × 3  m (height). Through quantitating the cell migration across the microchannels, the chip objectively reported how GBM cells migrate across tight spaces.

**Figure 3:**
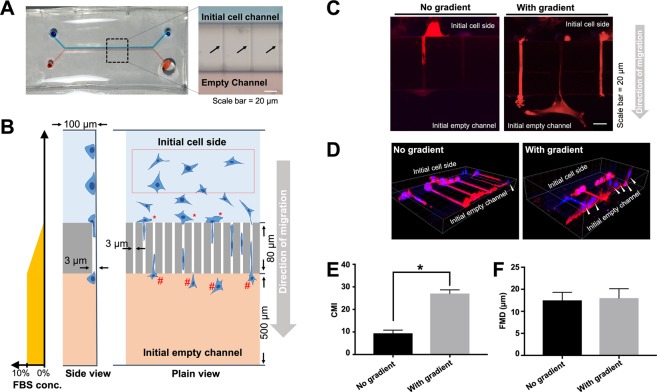
Schematic diagram and validation of migration chip for studying GBM cells migration. (**A**) Outlook of the migration chip. Red and blue dyes were injected to visualise two main channels with dimensions of 100 µm (H) × 500  m (W). The two channels were interconnected by microchannels (black arrow) with a cross-section of 3 µm (H) × 3  m (W). and length 80 µm. (**B**) Schematic illustration of the migration chip. Red box indicates U87 cells in the initial channel, where migrations was unrestricted. Motility was quantified and reported as free migration distance (FMD). “*” and “^#^” indicate cells that initiated migration, and completed migration across the microchannels, respectively. Cell migration was quantified and reported as cell migration index (CMI). The predicted FBS concentration gradient between the two main channels is illustrated in the plot to the left. (**C**) U87 cell migration across the microchannels was enhanced by a concentration gradient of serum across the microchannels. Cell protrusions and filopodia were more pronounced under this concentration gradient. (**D**) Successful migration of U87 cells was defined as a translocation of the nucleus across the microchannels. White arrows indicate migrated U87 cells. Blue is nucleus. Red is cytoplasmic mCherry. (**E**) Migration across microchannels, which is indicated by CMI. (**F**) Extent of U87 cells unrestricted motility, indicated by FMD (n = 3, error bar = ±1 SD. indicates p < 0.05).

The uniformity of the microchannels were first affirmed by imaging the fluorescence of Cy5-labelled collagen I, that was coated onto the walls of microchannels (Supplementary Figure 1C). Blue and red food dyes were then injected into two main channels, respectively, to confirm the structural integrity of the chip (Fig. 3A). The two-coloured food dyes were not mixed instantly in the chip, indicating that the small size of microchannels efficiently lowered the rate of passive diffusion. The rate of mixing between the two main channels was further estimated by measuring the diffusion of fluorescence-labelled dextran from one of the main channels to another. A serum concentration gradient is commonly used to drive chemotaxis-mediated unidirectional migration of U87 cells across the microchannels. Our results show that a concentration gradient could be maintained up to 7–8 h (Supplementary Fig. 2A). This allowed us to mimic tight brain parenchyma and chemotaxis of GBM cells (Fig. 3B).

To validate the use of this chip in studying GBM cells, we first investigated U87 migration in the presence of a serum concentration gradient. As shown in Fig. 3C, under the influence of the serum concentration gradient, U87 cells had more lamellipodia and filopodia extended toward the empty microchannels in comparison to cells in the absence of serum concentration gradient. However, we observed that some cell migration attempts were unsuccessful even though their filopodia protruded across the microchannels. Therefore, the number of translocated nuclei was then used as a definite quantification of successful migration. As shown in Fig. 3D, the number of nuclei translocated through microchannels was observably greater when a serum concentration gradient was applied to the system. Quantitatively, the cell migration index (CMI) under the influence of the serum concentration gradient was 2.9-fold higher than those without serum (Fig. 3E). These results indicate the serum concentration gradient induced the directional migration across microchannels, consistent with the findings demonstrated in other studies^38,39^. Supplementary Movie 1 shows glioma cells migration under low-magnification objective in time lapse.

While CMI quantified the U87 cell migration across confined microchannels, we also studied unconfined cell migration in the main channel (Fig. 3B, red box), which is reported as the “free-migration distance” (FMD) value. In contrast to CMI, there was no significant differences between the FMD values of GBM cells in the chips with and without serum concentration gradient (Fig. 3F). This indicates that the serum concentration gradient did not affect the motility of the cells that were not in close proximity to the microchannels. These results suggest that the microchannels in the migration chip are a good representation of GBM cell infiltration across a confined space similar to that in brain parenchyma and can serve as a physiological representative model for cell migration.

### Tf@pSiNP uptake prevent GBM to migrate through the microchannels

The migration chip was then applied to gauge the effect of Tf@pSiNP uptake on GBM cell migration. After seeding and incubation of GBM cells in the chip overnight, similar to the scrape-migration experiment, U87 cells were exposed to Tf@pSiNP, BSA@pSiNP, and Tf alone.

**Figure 4:**
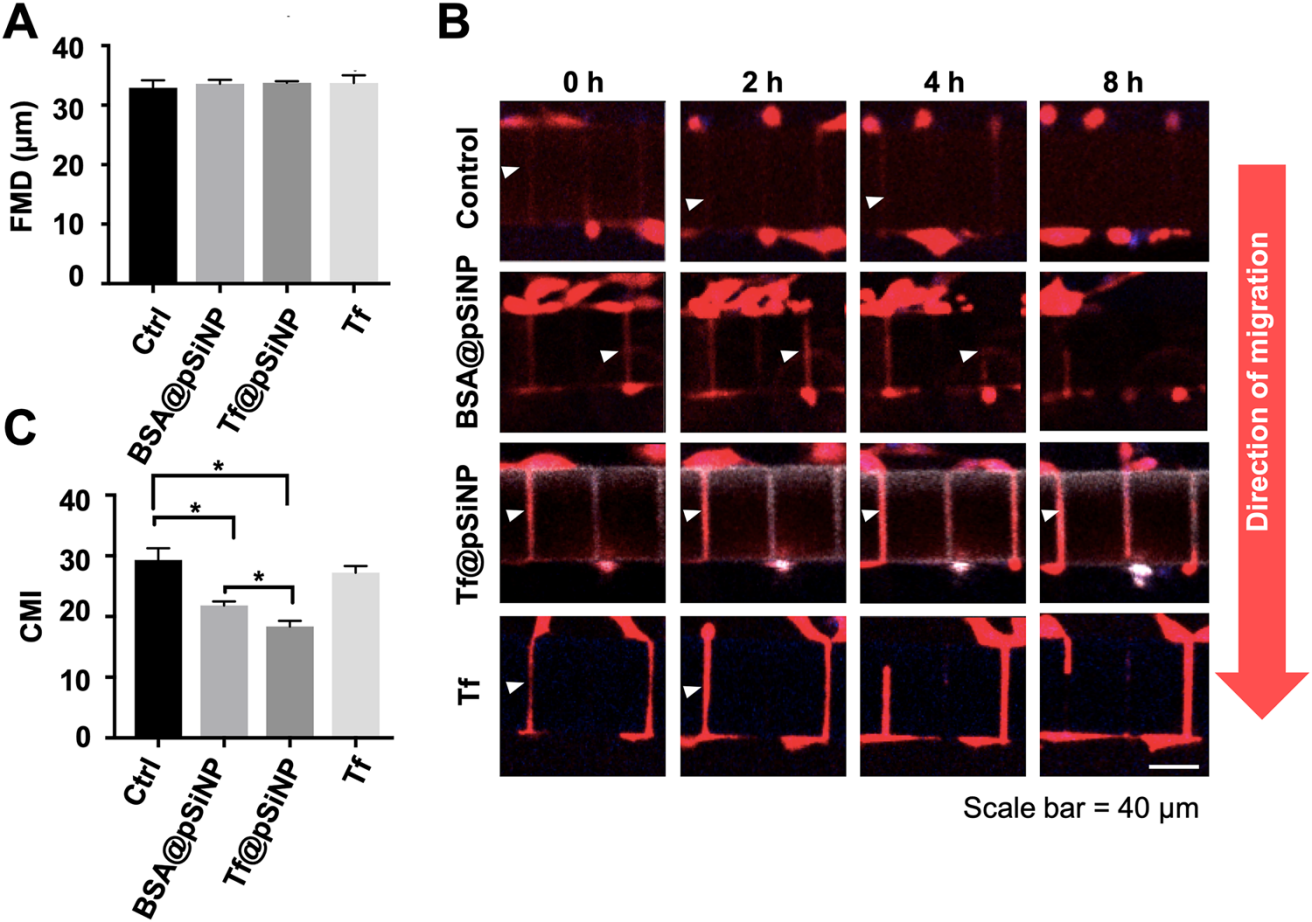
The effect of Tf@pSiNP, BSA@pSiNP and Tf treatments on GBM cells migration in the migration chip. (**A**) U87 cell motility (restriction free) in the initial cell side reported as FMD. FMD was averaged from measurements taken from 3 chip units (n = 3). (**B**) Montage of U87 cell migration across the microchannels after treatments over the period of 8 h. Red is cytoplasmic mCherry. (**C**) Cell migration across the microchannels reported as CMI (n = 3, error bar = ±1 SD. * indicates p < 0.05).

No significant differences were noted in FMD values of Tf@pSiNP-, BSA@pSiNP-, and Tf-exposed cells compared to untreated control group (Fig. 4A). These observations are in accordance with the scrape-migration assay result that cell migration was not affected by pSiNP exposures in the unconfined space.

The dynamic process of GBM cell migration through microchannels was captured via confocal time-lapse images (Fig. 4B). It was observed that a migrating U87 cell first extended a protrusion, the leading front of a cell, into the initial empty microchannels, while the retracting ‘tail’ was positioned on the initial cell side. We observed that this process, where a cell body was bridging across the microchannel, was an intermediate stage for cell migration. The intermediate stage was observed in all groups when GBM cells were migrating through the microchannels (Fig. 4B, white arrows). However, it was observed that the time required for a cell to protrude and retract its filopodia varied largely from cell to cell. Therefore, successful migrations were defined only by the translocation of nucleus from initial cell side to initial empty channel. Collectively, the rate of successful migration varied depending on the treatment. In contrast to the FMD value, we observed that Tf@pSiNP treatments altered the CMI value significantly (Fig. 4C). CMI value of Tf@pSiNP exposed cells lowered by 40% as compared to the control, indicating that Tf@pSiNP inhibited GBM cells migration. In contrast, there was no significant difference between the CMI of Tf and control, suggesting that Tf alone did not significantly inhibit migration through microchannels. These results suggest that the inhibition was due to the Tf@pSiNP internalisation, rather than the targeting molecule Tf. This is corroborated by the observation that the CMI of Tf@pSiNP exposed cells being significantly lower than those exposed to BSA@pSiNP, as well as the positive correlation with the level of internalisation (Fig. 1D).

We further investigated the effect of NP treatment exposure time on cell migration. We compared Tf@pSiNP exposure at 1 h and 8 h and found that there was no significant difference in CMI of GBM cells (Supplementary Figure 2A). This may suggest that a reasonably short Tf@pSiNP incubation time is sufficient to modulate GBM cell migration ability across microchannels.

**Figure 5:**
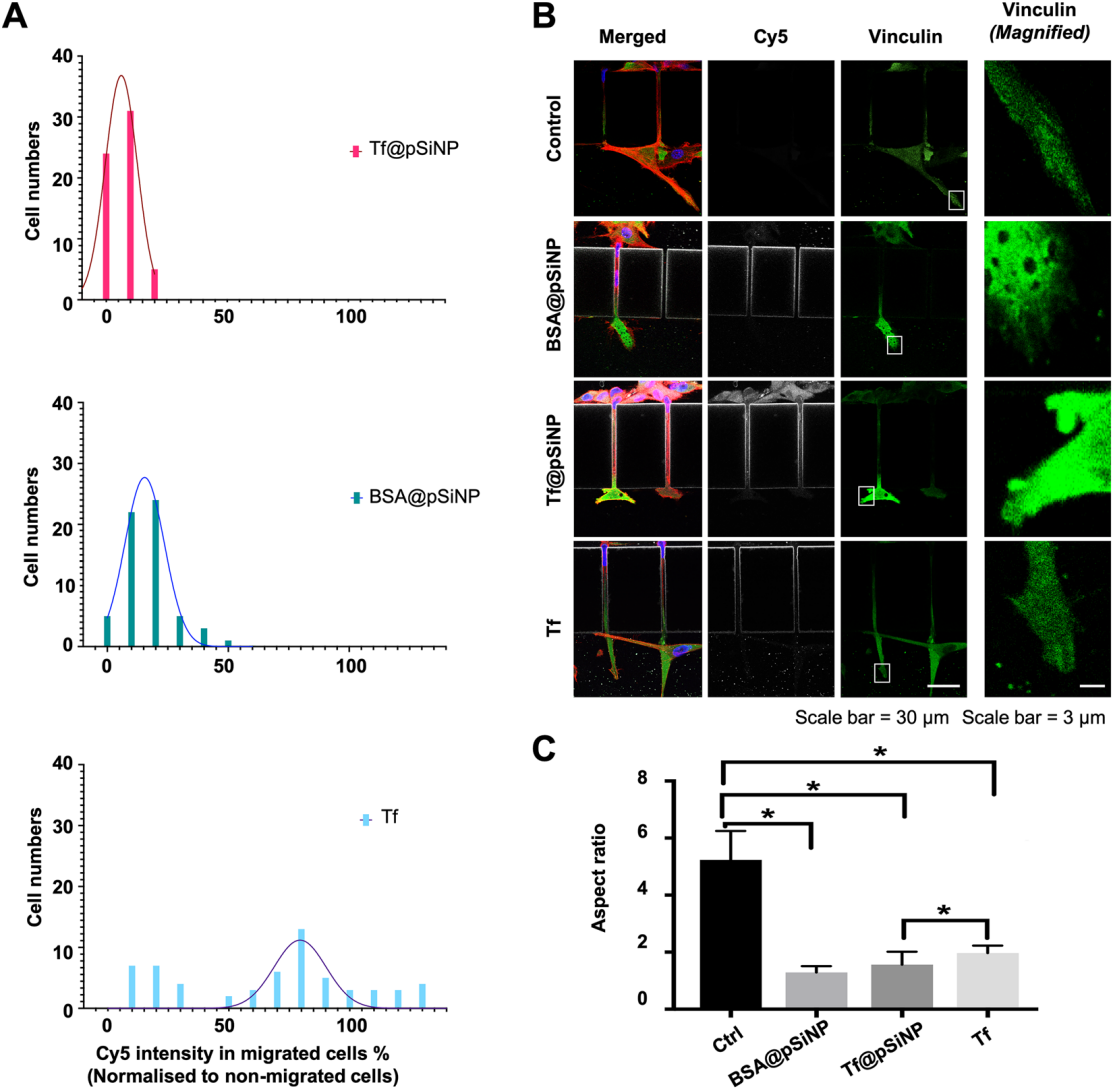
Visualisation of FA in U87 cells migrating across microchannels. (**A**) The amount of Tf@pSiNP, BSA@pSiNP, and Tf associated with U87 cells which migrated across microchannels as indicated by Cy5 intensity normalised to their respective non-migrated cells. Higher value indicates higher cell-pSiNP or cell-Tf associations in migrated cells. (**B**) Immunofluorescence staining of vinculin as a representation of FA. Filament actin, red. Nucleus, blue. Vinculin, green and white is Cy5-labelled Tf@pSiNP, BSA@pSiNP, and Tf. (**C**) Aspect ratio of identifiable FA in U87 cells. (n = 5, error bar = ±1 SD. * indicates p < 0.05).

Despite the observed inhibition of migration, 60% of cells treated with Tf@pSiNP which initiated migration through microchannels, succeeded. It was possible that the extent of cellular uptake of NP varied among the cells. There appears to be a correlation between cell migration and NP uptake. Tf@pSiNP, BSA@pSiNP, and Tf were labelled with Cy5 fluorescent reporter in order to visualise the relative quantity of cell association. To reveal the correlation between NP uptake and its impact on the ability to migrate through microchannels, we quantified and compared the associations between Tf@pSiNP, BSA@pSiNP, and Tf in migrated U87 cells (Fig. 5A). The successfully migrated cell population in Tf@pSiNP and BSA@pSiNP treated groups had distinctively lower Cy5 association than that of Tf treated group. This indicates that the cells, which successfully migrated across the microchannels, took up fewer particles as compared to the cells which did not migrate. In contrast, Tf treated cells showed less difference in uptake between migrated and non-migrated cells, again consolidating that Tf alone did not modulate cell migration across microchannels. This highlights that the extent of migration inhibition correlated to the level of pSiNP association.

### Focal adhesions are de-stabilised in migrating GBM cells treated with Tf@pSiNP

The unexpected migration-inhibitory effect of pSiNP on GBM cells prompted us to further investigate the mechanisms involved. As the coordination of focal adhesion (FA) formation is necessary for cell spreading and migration^40^, we stained for vinculin, an adaptor protein in FA, by immunofluorescence (IF) to reveal the impact of Tf@pSiNP on the maturation of FA.

Across all treatment groups, the first observation made was that vinculin staining was generally more pronounced in the filopodia extending across microchannels towards the empty channel side as compared to the cell bodies remaining on the initial channel side (Fig. 5B). This may reflect the polarisation of GBM cells during migration. Comparing all treatments, the vinculin staining intensity was higher in the filopodia of Tf@pSiNP- and BSA@pSiNP-treated cells. This indicates that more vinculin was expressed, and the subcellular location of vinculin was more polarised in those cells. Conversely, the vinculin recruited at the filopodia of control cells displayed defined rod shape structures. This is an indication of vinculin colocalising to talin that unfolds via mechanical force between cytoskeleton and integrins^41^. Notably, although cells treated with Tf@pSiNP and BSA@pSiNP expressed more vinculin at the filopodia, vinculin appeared as diffuse shape patterns rather than defined rod shape structures. This may suggest that vinculin was not recruited to reinforce the FA of those cells.

Furthermore, we analysed the aspect ratios of the FA (Fig. 5C). The data demonstrates that the FA of control cells had higher aspect ratio than those receiving pSiNP and Tf, indicating that the structure of FA in the control cell filopodia were more elongated. While the FA of cells receiving Tf@pSiNP and BSA@pSiNP were more circular in shape and were less defined. This might suggest that the FA in those cells were less established. And since FA is a critical apparatus for cells to gain traction during migration, our finding might explain at least in part the reduction in migration observed in Tf@pSiNP and BSA@pSiNP treated cells.

**Figure 6:**
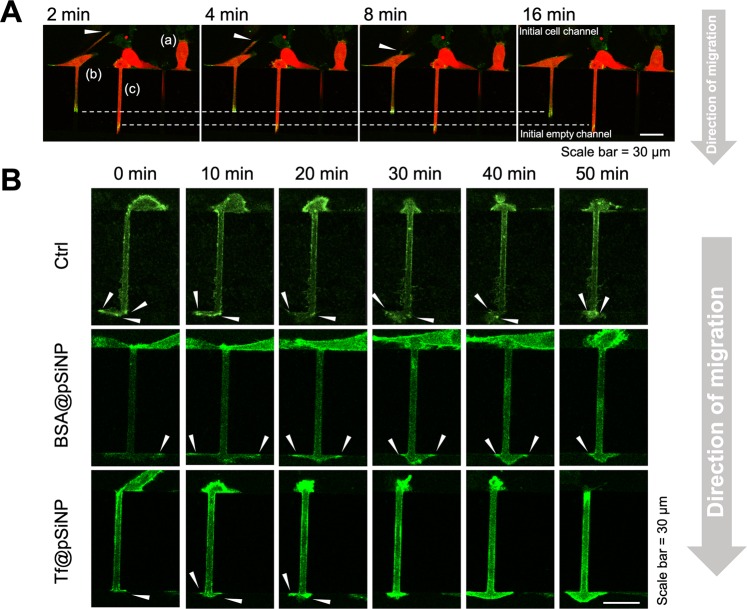
Time-lapse imaging of FA dynamics in U87 cells migrating across microchannels. (**A**) Stages of untreated cell migration across the microchannels. Cell (**a**) migrated to the entrance of microchannels; Leading front of cell (**b**) was protruding across the microchannels, while a tail was retracting (arrows); Cell (**c**) was at the intermediate stage where cell body spanned across entire microchannel. Cytoplasmic mCherry, red. Talin-GFP, green. (**B**) Talin-GFP expressing U87 cells treated by Tf@pSiNP, BSA@pSiNP, or left untreated, at their intermediate stages of migration across the microchannels. White arrows indicate observable FA at the leading fronts of the cells.

Since immunofluorescence (IF) only afforded a snapshot of the morphology of FA and did not resolve the effect of pSiNP exposure on the dynamics of FA formation, we transiently transfected talin-GFP into the cells. Talin is an adaptor protein that binds to vinculin during FA maturation and provides cytoskeleton-ECM traction^41^. We identified that U87 cells first migrated to the entrance of microchannels (see Fig. 6A, cell (a). Prior to formation of a protrusion of filopodia into microchannels, the talin expression was not prominent. Cell (b) represented the next stage of migration where the leading front protruded into the microchannel, while a tail was retracting (arrows). Cell (c) represented cells at the intermediate stage where the cell body spanned across entire microchannel before it had completed the migration across the channel. We frequently observed that a cell oscillated back and forth during this intermediate stage before completing translocation. Supplementary Movie 2 shows the dynamics described in Fig. 6A in time lapse.

Figure 6B shows time-lapse images of talin-GFP expressing cells treated with Tf@pSiNP, BSA@pSiNP, and the untreated control. All cells shown here were at the beginning of the intermediate stage of migration across the microchannels, where the leading front and the retracting tail of the cells could be identified. At the time of 0 min defines as the start of the intermediate stage, where the leading front had reached the empty channel and displayed mature FA. For all cells, a greater number of defined FA was present in the leading fronts (FA indicted by white arrows) in comparison to the retracting tail. There were more clearly defined FA in the leading front of the control cells and BSA@pSiNP exposed cells compared to Tf@pSiNP treated cells at the 30 min lapse time. The differences widened as the migration progressed, and the originally well-defined FA in both of the pSiNP treatment groups became diffuse. This observation was consistent with our IF results and confirmed the difference existing at the leading front of the cells, which may partially explain the observed reduction in migration.

### Inability of GBM cells to shrink plays a role in the Tf@pSiNP-mediated migration inhibition

The impact of Tf@pSiNP uptake on FA dynamics at the leading front of migrating U87 cells gave us a possible explanation for the reduced migration propensity. Interestingly, comparing Tf-treated and Tf@pSiNP-treated cells, both displayed less-established FA, while only Tf@pSiNP caused significant reduction in CMI (Fig. 4C). This indicated that Tf@pSiNP treatment modulated GBM migration as well as through other mechanisms. In addition to FA dynamics, it is well-known that reduction in cell volume is also a critical step for brain cancer cells to navigate narrow brain parenchyma^9^. This led to study the effect of Tf@pSiNP uptake on the ability of U87 cells to undergo volumetric changes.

**Figure 7:**
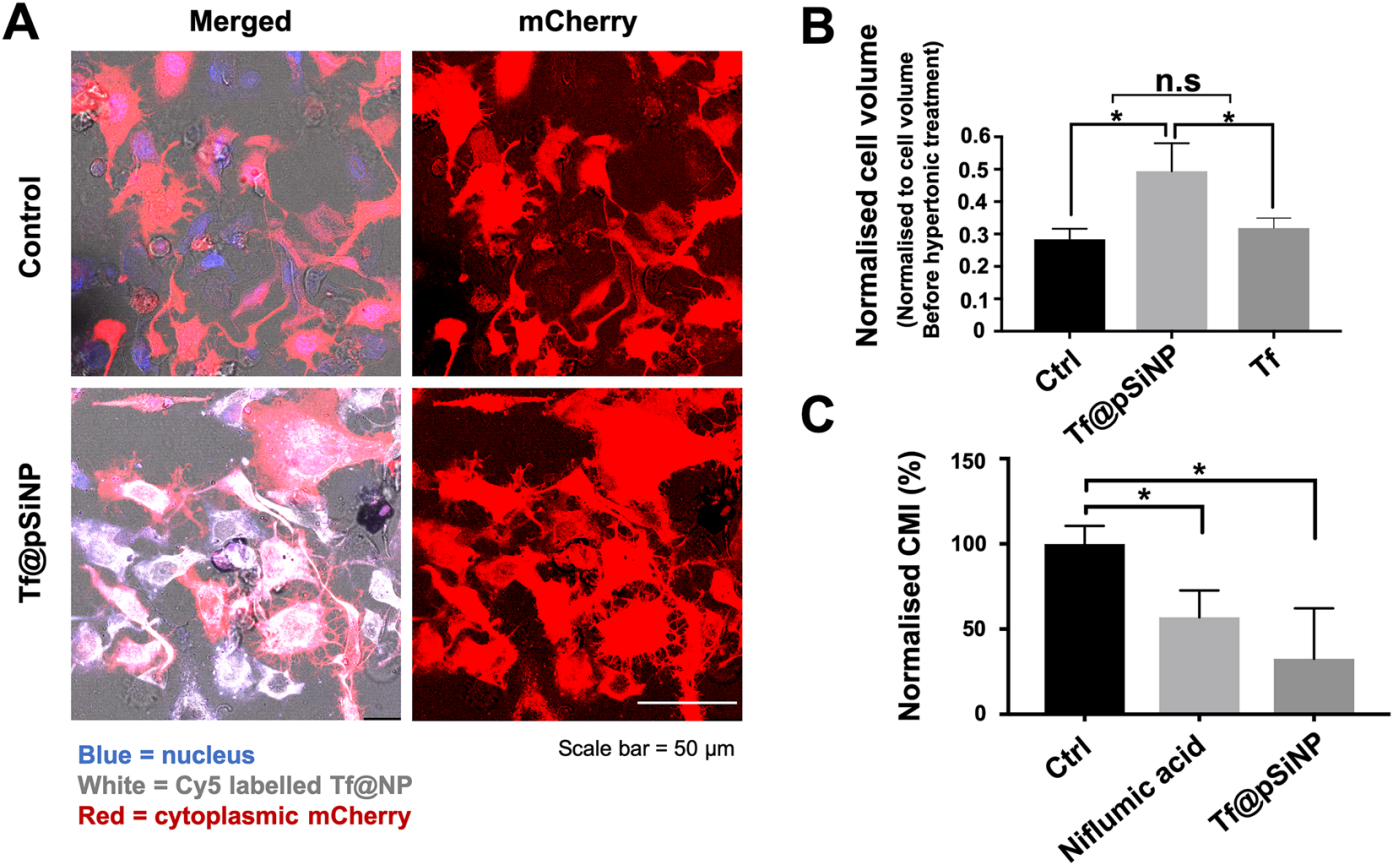
Characterising the effect of Tf@pSiNP on GBM cell volume regulation and cell migration. (**A**) Morphology of U87 cells exposed to hypertonic medium imaged by means of scanning confocal microscopy. Cytoplasmic mCherry, Red. Cy5-labelled Tf@pSiNP, white. Blue corresponds to nuclei. (**B**) Normalised U87 cell volume exposed to hypertonic medium. Cell volume measured is normalised to the cell volume measured before hypertonic treatment. A smaller normalised cell volume indicates larger cell volume reduction under hypertonic treatment. (n = 3, error bar = ±1 SD. * indicates p < 0.05). (**C**) Quantification of cell migration index of U87 cells treated with Cl^−^ channel blocker (niflumic acid) and Tf@pSiNP.

After exposure to Tf@pSiNP for 8 h, cells decreased their cell volume under osmotic pressure induced by the hypertonic medium (1 osmol/L sucrose in DMEM). Fluorescence microscopy images of cells treated with Cy5-labelled Tf@pSiNP indicate that the cells had taken up Tf@pSiNP (Fig. 7A). Under confocal z-stack imaging, these cells had a larger cytoplasmic mCherry volume compared to control group cells, indicating that cells treated with Tf@pSiNP were seemingly larger in size than that of the control cells under hypertonic osmotic pressure. We quantified the extent of the reduction in cell volume by normalising it to that before hypertonic medium treatment, and the ‘cell volume change’ was reported. Our results show that Tf@pSiNP-exposed cells reduced cell volume by 51% while untreated cells reduced cell volume by 72% (Fig. 7B). This confirms that Tf@pSiNP treatment decreased the ability of GBM cells to response to osmotic pressure and subsequent cell volume reduction. In addition, Tf exposed cells (without pSiNP) under hypertonic medium also reduced cell volume by 68%, indicating that Tf treatment alone was unlikely to mediate the resistance of cells responding to osmotic pressure. Since we observed that cytoplasmic water was released from control cells almost instantly (1 min) after inducing the hypertonic medium, the process was possibly facilitated by aquaporin water channels^42^.

The fact that Tf@pSiNP treatment resists GBM cell volume reduction prompted us to study how cellular volume reduction affects migration through the microchannels of our migration chip. Ion channel blockers, such as niflumic acid, are well-known to inhibit GBM cell volume reduction, and are being studied as anti-metastatic agents^43^. Therefore, we first studied the capability of niflumic acid-treated U87 cells in migrating across microchannels. We observed that the CMI of Cl^-^ channel blocker niflumic acid-treated U87 cells significantly dropped by 43% as compared to that in untreated cells (Fig. 7C). This indicates that the migration across microchannels in the chip required a reduction of the cell volume. Interestingly, we also observed that CMI of cells treated by Tf@pSiNP was not significantly different to those cells treated by niflumic acid (Fig. 7C). To validate these observations, a more clinically relevant primary glioma cell (WK1) had been treated with Tf@pSiNP and niflumic acid, and their migration were evaluated using a conventional Transwell model with the same pore size of 3 µm (Supplementary Fig. 2C). The results indicate that Tf@pSiNP and Cl^-^ channel blocker exposures were also able to inhibit the migration of primary glioma cells, confirming our observations using U87 cells and our migration chip. The data collectively suggests that Tf@pSiNP was likely inhibiting the migration of GBM cells by impacting the ability to reduce cell volume.

## Discussion

It is known that 90% of cancer mortality is a result of metastasis^44^. Inhibiting or eliminating cancer metastases is an obvious approach to improve survivability. Although cancer nanomedicine is more commonly driven to realise drug delivery and diagnosis, there has been sporadic evidence that some nanoparticle carriers (NP) alone can influence the migration of cancer cells. For example, Wang *et al*. demonstrated that ursolic acid NP inhibited cervical cancer migration through inducing apoptosis^21^. BSA-based GRP78 receptor-targeting NP have been shown to inhibit hepatocellular carcinoma (HCC) cell invasion via receptor-mediated binding^20^. While local metastasis is the hallmark and the root cause of the high brain cancer mortality, there has not been any attempt so far as published in public domain to study the effect of NP uptake on invasion.

To address this research gap, we used pSiNP as a model NP due to its excellent biocompatibility and drug encapsulating capacity. Since TfR is overexpressed on GBM cells and on the luminal side of blood-brain barrier (BBB)^27,45^, it has become a popular target in a plethora of drug delivery studies. For example, Tf has been widely explored as a targeting ligand for improving BBB penetration of nanocarriers such as micelles^46^, liposomes^47^, and polymersomes^48^. Coating of Tf on pSiNP through hydrophobic interactions was first described by Reuter *et al*.^49^ They observed that Tf modification enhanced the internalisation into breast cancer cells. In this work, we studied whether Tf conjugation on pSiNP surface could enhance the internalisation into GBM cancer cells. Although pSiNP was mostly plate shaped, unlike spherical shaped liposomes and gold NP, we can demonstrate that Tf-decoration could result in a 3 fold increase pSiNP uptake into GBM cells compared to non-targeting BSA-coated pSiNPs (Fig. 1D). This enhancement is similar to those reported for gold NP decorated with Tf^50^.

As predicted, Tf@pSiNP were non-toxic and did not reduce ATP amount in GBM cells, in agreement to other pSiNP studies *in vitro* and *in vivo*^23,51^. In a separate study, we also showed that Tf@pSiNP is also highly tolerable in human cerebral microvascular cells, and non-CNS keratinocytes over a wide range of concentration^35^, highlighting the biocompatibility of those nanoparticles. Since the cells exposed to Tf alone were also highly viable, the amount of Fe^2+^ released from Tf did not cause detrimental effects such as ferroptosis^52^. Therefore, we believe that Tf@pSiNP was a suitable model NP for us to study how Tf@pSiNP treatment would impact on the ability of GBM cells to migrate.

Our “scrape-migration” assay result showed Tf@pSiNPs had no migration inhibition in GBM cells. However, it should be noted that this conventional assay studies cells migration on a flat culture surface without any topographical restrictions, which is highly different to the brain parenchyma. Indeed, GBM cells diffusively infiltrate to the healthy brain tissue along narrow extracellular routes, such as stiff neuronal tracts, which require substantial cytoplasmic volume changes and highly regulated FA dynamics^43,53^. These restrictions are commonly mimicked in a Transwell model, in which cancer cells migrate through narrow pores of a polymeric membrane. And the model has been used to gauge changes in cell migration. However, opaque Transwell membranes are not compatible with high-content imaging of morphological changes and FA dynamics during migration, which is crucial to study the impact of Tf@pSiNP on cancer cell migration.

To overcome these limitations, we applied a microfluidic chip with tight topographical restrictions, in the form of an array of 3 μm × 3 μm microchannels connecting two parallel channels to study GBM cell migration. Such side-by-side placement of optically clear microchannels allowed simultaneous time-lapse visualisation of cells and direct measurement of the dynamics of the migration processes, which is a significant advantage as compared to the Transwell model^54^. Indeed, similarly designed microchannels have been used to study the effect of different drugs or chemoattractant on migration rate of various cancer cells^55^. Microchannel system was also applied to isolate brain tumour stem cells (BTSC)^56^, and to study the mechanical properties of migrating GBM cells under confinement^57^. However, to the best of our knowledge of published literature in the public domain it has never been applied to study the effect of NP treatment on GBM migration ability.

Although 8 μm diameter pores in the Transwell being widely used for cell migration studies^58,59^, GBM cells were shown capable to migrate through even smaller restrictions. For example, a study by Harada *et al*. demonstrated that GBM cells migrated through pores of 3 μm diameter 35-fold faster than other cancer cells^60^. In the current study, we also found that human GBM cells U87 were able to migrate through the 3 μm microchannels of our migration chip under serum concentration gradient as a chemoattractant.

Specifically, we visualised the migration of GBM cells in a time-resolved manner. It was observed that the GBM migration through a microchannel was initiated by sending leading front cell protrusion across the microchannel. An intermediate stage followed suit, where the cell body spanned across the entire microchannel with the leading front protrusion on the new channel side, while the retracting tail and the nucleus still remained in the original channel. Successful migration of GBM cells depended on whether the nucleus could be translocated and the tail could be retracted through the microchannels. This result in principle agrees with a study by Monzo *et al*.^61^, in which the two phases of GBM cell migration down a track of 4 μm width were described, including cell elongation and tail retraction. The authors also reported that migrating GBM cells were polarised, where FA at the leading front were mostly defined and stationary, while the retracting tail FA were less established. Consistent with this report, our vinculin staining for FA also showed polarised morphologies. However, the migration speed of cell bodies reported by Monzo *et al*. was around 50 μm/h, while in our study most U87 cells took 8 h to complete the translocation across the microchannels (10 μm/h). Another study by Prahl *et al*. reported that U251 migrated in an enclosed linear channel (12 μm width, 5 μm height) at a speed of 30 μm/h^57^. Since the microchannels that we used had a smaller cross-section (3 μm width, 3 μm height, Fig. 3), we believe that the differences may due to the level of confinement.

Migration and invasiveness of GBM cells are emerging as areas of therapeutic focus. In fact, small molecular ion channel inhibitors, such as chlorotoxin derivative TM601, blocker of Kv10.1 antihistamine and astemizole, were sought as ways to target GBM invasion^62^. We studied the effect of Tf@pSiNP treatment on GBM cell migration across microchannels and discovered that treatment with Tf@pSiNP reduced GBM cell migration with the extent of reduction correlating positively to the extent of pSiNP-cell association, while this effect was absent for cells treated with Tf (Fig. 4). There were existing studies describing the inhibition of invasion by using NP loaded with radioisotopes and chemotherapeutics on hepatocellular carcinoma and gastric cancer^20,63^, in which the cell invasions were inhibited by the cytotoxicity of the payload. In this study, pSiNP was not carrying any therapeutics, and the treatment did not hamper viability nor intracellular ATP content (Fig. 2). Therefore, the result obtained here was encouraging and intriguing.

Understanding this effect is important to translate such observation into clinical applications. We found that the FA at the leading front of migrating U87 cells, which were treated by Tf@pSiNP, were morphologically less mature as compared to untreated cells (Fig. 5B). In addition, the observed elongated morphology is an indication of better cell migration ability^64^. Increased vinculin localisation at elongated FA is a result of force-triggered talin unfolding and talin-vinculin binding, a traction force that is needed during cell migration^65,66^. Therefore, we believe that the internalisation of pSiNP may attenuate the maturation of FA, which in turn hampers migration in U87 cells. However, since the FA of U87 cells exposure to Tf alone also displayed less elongated morphology as compared to control, we speculated that the attenuation of FA may only be one phase of the overall mechanisms.

The fact that pSiNP did not impede cell migration in a non-confined migration setting (scrape-migration assay), but in a microchannel migration setting, prompted us to investigate and discover that Tf@pSiNP internalisation diminished the ability of GBM cells to reduce cell volume (Fig. 7A,B). Our data also suggests that successful cell migration through the tight microchannels in our migration chip required cell volume reduction (Fig. 7C). This agrees with long-standing observation that GBM cells dramatically reduce cell volume through extrusion of cytoplasmic water upon migrating along confined and mechanically rigid barriers, such as the vasculature’s abluminal surface or white matter tracts^67^. Together they suggest that the disabling of GBM cells to ‘shrink’ could explain the reduced cell migration across confined microchannels. Importantly, we observed that untreated GBM cells responded within 1 min to hypertonic pressure by shrinking while pSiNP-treated ones did not, indicating a possible involvement of aquaporins that facilitate rapid water efflux at a much higher rate than passive diffusion across the plasma membrane^42^. Another possible explanation that we cannot exclude at this point is the physical ‘crowding’ of the cytoplasm with incompressible NP. In fact, Ali *et al*. made the similar observation that nuclear membrane-targeting gold NP inhibited ovarian cancer cell migration by physically increasing the stiffness of the nucleus^17^.

**Figure 8:**
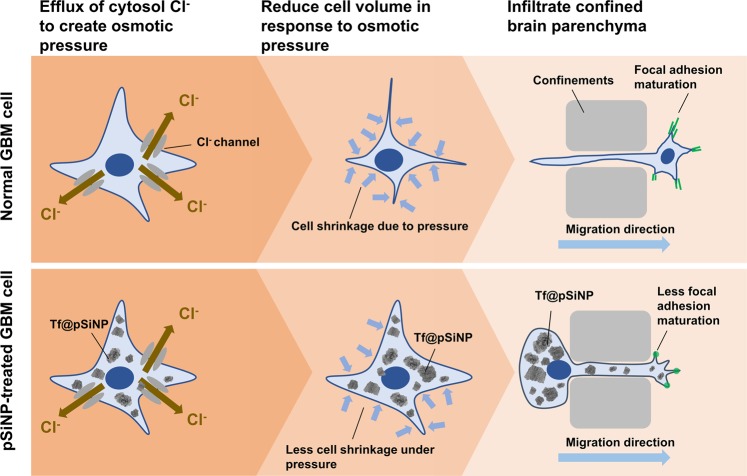
Mechanism of cell volume reduction in GBM cell migration across a confined space, and the proposed effect of pSiNP treatment on cell volume reduction.

Our data supports the hypothesis that pSiNP attenuated cell migration by resisting cell volume reduction in response to osmotic pressure, and the destabilisation of FA may have also contributed to the effect (Fig. 8). Since this proposed mechanism is fundamentally different from some well-established metastasis inhibitors, which disable Cl^-^ ion channels to drive osmotic pressure difference^9^, the combined application of both may further hamper the cell migratory ability. Future studies should, thereby, explore the clinical potential of such combined treatment. Although the mechanism of how pSiNP attenuated GBM cell volume reduction and focal adhesion maturation remains to be further clarified, this work reported pSiNP as a new tool that is potent in reducing GBM cell migration and might enable new treatments option for GBM patients.

In this study, we developed a GBM cell-targeting nanoparticle, Tf@pSiNP, and studied its migration inhibitory effect on a microfluidic-based migration chip. This platform better mimicked the tight extracellular migration tracts in brain parenchyma, and allowed high-content time-resolved imaging of GBM cell migration. We have shown that Tf@pSiNP were hydrodynamically stable, biocompatible, and their cellular uptake into GBM cells was enhanced by Tf surface functionalisation. GBM cells exposed to Tf@pSiNP were shown less capable to migrate through tight microchannels. The level of inhibition was dependent on the uptake of the pSiNP core, but not the targeting molecule Tf. The pSiNP-induced destabilisation of FA at the leading front may partially explain the migration inhibition. Additional corroborating evidence suggests that pSiNP uptake reduced the plasticity of GBM cells facilitating cell volume reduction, which is essential for the migration through tight confined tracts. We believe that the inhibitory effect of Tf@pSiNP on cell migration, together with the drug-delivery capability of pSiNP could potentially lead to the realisation of a disruptive strategy to treat GBM.

## Materials and Method

### Cell culture and maintenance

Human U87 cells that stably express both intracellular mCherry and firefly luciferase, donated by Bakhos A. Tannous, MGH, Massachusetts, were cultured at 37 °C in 5% CO_2_ atmosphere in a humidified incubator. We chose U87-mCherry cells as a model for this study as the cytoplasmic mCherry expression allows the tracking of cell body under time-lapse cell imaging. The mCherry reporter expression and firefly luciferase activity in the U87 cells were clearly observable under microscopy and under *in vivo* bioluminescence imaging (data not shown), respectively, in agreement with the existing documentations characterising the cell line^68,69^. This cell line was tested negative for mycoplasma contamination by us using PlasmoTest^TM^ Mycoplasma Detection Kit (InvivoGen, cat rep-pt1). For experiments that did not require live-cell tracking cell body, such as endpoint immunofluorescence experiments and talin-GFP experiment, wildtype U87 was used. For both cell types, Dulbecoo’s Modified Eagle’s Medium (DMEM) high glucose (Gibco, 11995073) supplemented with 10% heat inactivated fetal bovine serum (FBS, Invitrogen), 1% antibiotic-antimycotic (Anti-Anti, Invitrogen) was used to culture the cells. Culture media were refreshed every three days and the confluency was maintained below 80%.

A validation experiment was conducted on patient-derived glioblastoma WK1 cells, which were provided by Bryan Day, QIMR, Brisbane, Australia. The phenotype and heterogeneity of the cells were previously characterised^70,71^. Cells were cultured in knockout Dulbecco’s modified Eagle’s medium (DMEM, Gibco, 12660-012), supplemented with 0.1 mg/mL recombinant human Epidermal Growth Factor (Gibco, PHG0314), 0.05 mg/mL recombinant human Fibroblast Growth Factor basic (Gibco, PHG0024), and StemPro Neural Supplement (Gibco, A10508-01). The culture media also contained GlutaMAX (Gibco, 35050-061) and penicillin/streptomycin (Gibco, 15140-122). Cells were only used between passages 16 and 25. Cells were passaged at approximately 90% confluency by incubating in Accutase solution (Sigma-Aldrich, A694), followed by inactivation with trypsin inhibitor. This primary cell was also tested negative for mycoplasma contamination.

### Porous silicon nanoparticle (pSiNP) preparation

pSiNP was fabricated according to a previously reported procedure^23,35^. p+ type (0.01–0.02 Ω cm) silicon wafers (Siegert Consulting Co., Aachen, Germany) etched periodically at 50 (2.2 s period) and 200 (0.35 s period) mA/cm^2^ in a solution of 1:1 HF (38%):ethanol (EtOH) for 20 min. Afterwards, current density was increased to detach the porous silicon (pSi) films from the substrate to electropolishing conditions (250 mA/cm^2^, 3 s period). Then, pSi multilayer films were thermally hydrocarbonised under N_2_/acetylene (1:1, v/v) flow at 500 °C for 15 min and cooled down to room temperature in a N_2_ gas flow. The films were then immersed into neat undecylenic acid at 120 °C for 16 h. After this, the COOH-functionalised films were milled down into NP by ball milling in a 10 vol-% undecylenic acid-decane mixture. The obtained particles were washed with ethanol to remove the milling media. The size selection of the NP was done by centrifugation in ethanol and the final UA-functionalised particles (UnpSiNPs) were stored in ethanol at 4 °C for further use.

### Preparation of covalently bound Tf /BSA@pSiNP

Human holo-transferrin (Sigma T4132) was covalently bound to the UnpSiNP via EDC/NHS reaction, and the end product was termed Tf@pSiNP. Briefly, UnpSiNP were washed three times with (2-N-morpholino) ethanesulfonic acid buffer (MES, pH 6.0) to remove the EtOH, and resuspended into 0.5 mL MES buffer after final wash (final concentration 10 mg/mL). 0.5 mg of 1-ethyl-3-(3-dimethylaminopropyl)carbodiimide (EDC) hydrocholoride (Sigma 03459, final concentration 2.6 mM) and 1.1 mg of N-hydroxysulfosuccinimide (sulfo-NHS, Sigma 56485, final concentration 5 mM) were added to the reaction. The reaction components were mixed well and allowed to react for 15 min at room temperature. After the NHS ester activation, MES buffer was replaced by 300 µL PBS (pH 7.4). Then the suspension was added dropwise to 200 µL of transferrin solution in PBS (final concentration 10 mg/mL). The solution was mixed well and allowed to proceed for 2 h at room temperature with agitation. Then the reaction was quenched by 50 mM Tris for 15 min. Free transferrin was washed away by washing nanoparticles with PBS three times after the second step of amine reaction. The Tf@pSiNP were stored in PBS for further studies. BSA-functionalised nanoparticles were prepared in the same way as Tf@pSiNP.

### Dynamic light scattering and zeta potential measurement

To study the hydrodynamic size and zeta-potential of nanoparticles, the particles were washed and resuspended in PBS. After 2 min sonication, the particles were then analysed using a Malvern Zeta-sizer (Malvern, Worcestershire, UK). Scattering angle θ = 173° was chosen, and the analysis was carried out at 25 °C. The data shown herein are an average of at least triplicated measurements.

### Cryo-TEM imaging of nanoparticles

The Cryo-TEM was conducted following our previously described protocol^35^. Briefly, a 3 µL sample of NP in PBS was dispensed onto a glow discharged copper grid (300 mesh) with lacey carbon film coating (ProSciTech, QLD, Australia). The grid was blotted against filter paper (Whatman 541) and plunged into liquid ethane using an in-house plunge freezing device in 80% humidity. The samples were presented to the transmission electron microscope TEM (FEI, Eindhoven, The Netherlands) operated at 120 kV using a Gatan 626 cryo-holder (Gatan, Pleasanton, CA, USA). A low electron dose of 8–10 electrons/Å^2^ was employed. Images were captured using a FEI Eagle 4kx4k CCD camera (FEI) and AnalySIS software v3.2 (Olympus.).

### TEM visualisation of pSiNP internalisation

U87 cells were cultured in a 6-well culture plate at 37 °C and 5% CO_2_ humidified atmosphere, with a starting density of 25,000 cells per cm^2^. Cultures were maintained for 24 h prior to incubation with nanoparticles at a concentration of 0.1 mg/mL. Controls (untreated cells) were generated in the same condition by incubating U87 cells in medium but without nanoparticles. After 24 h, the cells were washed to remove the non-internalised nanoparticles. The preparation of TEM samples was conducted according to the protocol described by Luo *et al*.^35^. Briefly, the cells were first harvested by trypsinisation, and pelleted at 200 rcf. The cell pellets were fixed by glutaraldehyde treatment (3% in Sorenson’s phosphate buffer, pH 7.4, ionic strength 0.154 M) (ProSciTech, Thuringowa, QLD) at room temperature for 2 h, buffer washed, and post-fixed in 1% osmium tetroxide for 1 h. After washing, pellets were then gradually dehydrated in increasing ethanol concentration and transferred to propylene oxide. They were then infiltrated and embedded in Epon Araldite(RroSciTech, Thuringowa, QLD) polymerised at 55 °C for 48 h. 200-mesh copper grids were used to collect ultrathin sections, which were then stained with 8% methanolic uranyl acetate, followed by Reynold’s lead citrate, each for 5 min using conventional methodology. Samples were viewed by using a Tecnai T12 TEM (FEI, Eindhoven, the Netherlands) at an accelerating voltage of 120 kV.

### Fabrication of migration chip with microchannels

The chip was fabricated via photolithographic and soft lithographic techniques. A silicon wafer was used to fabricate the master mould via deposition of photosensitive polymer (SU8). Dual layer fabrication of SU8 was performed to resolve the first thin layer (3 μm) array of parallel microchannels connecting the second thicker layer (100 μm) of two main channels. The master mould was then replicated onto Sylgard 184 polydimethylsiloxane (PDMS) prepared by mixing at a 10:1 ratio (monomer to catalyst). It was poured onto the wafer to the height of 100 µm in a 150 mm Petri dish and degassed for 15–30 min. Then, the polymer was cured inside an oven at 80 °C for a minimum of 1 h. The cured PDMS mould was then detached from the developed master. The inlet and reservoir holes were punched using 1.5 mm and 2.0 mm Haris Uni-Core biopsy punches (Ted Pella), respectively. Glass slide (MENZEL-GLASER) was washed in water and ethanol once each. The bonding surfaces -glass slide and PDMS - were then cleaned and activated by oxygen plasma for 30 s (Harrick Scientific, Ithaca, NY). After assembly, the chips were further incubated at 80 °C for 15 min. Before using the chip, it was sterilised under UV (wavelength: 254 nm) irradiation for 30 min. To enhance the U87 attachment to the chip, the channels were treated with poly-L-lysin solution (Thermo Fisher Scientific) for 1 h at room temperature, then washed with PBS and DMEM, respectively.

### Characterisation of microchannels in migration chip

In order to visualise the main channels and microchannels in the chip, cyanine-5 (Cy5) dye labeled collagen was injected and probed the internal surface of the channels. Collagen type I from rat tail (1 mg/mL, Sigma-Aldrich 11179179001) was diluted in PBS and labeled with Cy5-NHS ester (Lumiprobe, 43020) by following manufacturer’s procedure. The resulting product Cy5-labeled collagen (100 μg/mL) in PBS was then injected into both main channels of the chip and incubated for 1 h at room temperature. The remaining Cy5-labeled collagen solution was aspirated and the channels were washed with PBS. The collagen-coated chip was then imaged under a confocal laser scanning microscope (Leica SP8).

### Cell seeding into migration chip with microchannels

To assess the potential of GBM cells in migrating through microchannels to mimick brain parenchyma, U87-mCherry or U87WT were first seeded and allowed to attach and acclimatise for at least 8 h prior to experimentation. Before the experiment (24 h), cells were labeled with live-cell nucleus tracker DRAQ5^TM^ (Invitrogen 62252) for 5 min such that the nucleus could be traced. Hoechst (33342) was used instead for experiments that required the visualisation of Cy5 conjugated pSiNP or Tf. Cells were then injected at a concentration of 10 ${\rm{\times }}$ 10^6^ cells/mL. Each channel held approximately 8 µL of medium and thus the cell seeding was consistently at 8 ${\rm{\times }}$ 10^4^ cells per channel. During the acclimatisation stage, no FBS gradient was applied across the microchannels (that is DMEM with 10% serum was used in both the cell channel and empty channel). According to our observations, the pre-experiment migration without serum gradient was minimal (Fig. 3F). The pre-experiment migration was subtracted from calculations of cell migration index to reveal the migrations only within the period of experiment.

### Immunostaining and transfection

Immunofluorescence conducted was based on the protocol recently described by Tang *et al*.^72^. The cells were cultured in the migration chips for 24 h, followed by treatment and migration for 8 h then fixed in freshly prepared 4% paraformaldehyde (PFA) at room temperature for 15 min. The cells were washed with PBS twice (5 min each time) and permeabilised in 0.1% Triton X-100 in PBS for 2 min. After washing with PBS twice (5 min each time), the cells were blocked with 1% bovine serum albumin (BSA) in PBS for 30 min. Primary antibody incubation was conducted overnight, the cells were then washed three times with PBS (5 min each time). Fluorochrome-conjugated secondary antibody incubation was conducted for 60 min. The primary antibody used was mouse anti-vinculin (1:200, Thermofisher, MA5-11690) and the secondary antibody used was Alexa 488 goat anti mouse (1:200, Invitrogen, A11001). After antibody incubations, the cells were washed twice with PBS (5 min). The nucleus and cytoskeleton were counter stained with Hoechst 33342 (Sigma Aldrich, B2261) and rhodamine-phalloidin (Invitrogen, R415), respectively. The stained samples were imaged with a confocal laser scanning microscope (Leica SP8).

To visualise the focal adhesion dynamics, plasmid encoding talin-GFP was transiently transfected into U87WT cells. Briefly, 300,000 cells per well were seeded into a 24-well plate. After 3 hours, CellLight® Talin-GFP, BacMam 2.0 transfection reagent (Thermofisher, C10611) were added to the culture reaching a ratio of 40 particles per cell (PPC). Then, transfected cells were incubated at 37 °C humidified incubator for 16 h before seeding in the migration chip.

### Time-lapse live-cell confocal microscopy

The migration of GBM cells after NP exposure were imaged over a time course of 8 h by using time-lapse confocal microscope. Briefly before the experiment, the initial cell channel in the migration chip (Fig. 3B), which was seeded with U87-mCherry cells, were gently perfused and replaced with CO_2_ independent medium (Thermo Fisher Scientific, 18045-088) with 0% serum containing pSiNP, Tf, or no treatments. The other channel was replenished with fresh CO_2_ independent medium with 10% serum. The migration chip was then immediately transferred to laser scanning confocal microscope (Nikon Ti-E and A1R) equipped with humidified culture chamber maintained at 37 °C environment. Images were captured every 20 min. To image focal adhesion (FA) dynamics, migration chip with U87WT transiently transfected with talin-GFP was treated similarly as described above, while the talin dynamics were imaged every 7 min.

An independent experiment was conducted to evaluate how the duration of exposure affects GBM cells migration. The imaging procedure remained unchanged while the unbounded treatments were washed off by perfusing fresh CO_2_ independent medium with 0% serum before imaging. Over all experiments, the extent of migration was analysed by counting the number of cell nuclei clearing the microchannels and reaching the initially empty channel. Details of image analysis are described in the image analysis section.

### Quantitative image analysis

U87 cell migration time-lapses (.nd2 format) of each experiments were imported to image analysis software Imaris ×64 (ver. 9.1.1 Jan 29 2018) to conduct analysis objectively. Using this software, we compare the cell motility and infiltration potential of U87 cells under different treatments based on two measurements, namely free migration distance (FMD), and cell migration index (CMI), respectively. For the analyses of Cy5 fluorophore intensity, and measurement of FA dimensions, Fiji ImageJ (ver 2.0.0) was used.

FMD (with the unit µm) is defined as the average distance travelled by individual cells over the period of the experiment. The higher the FMD indicates the population of cells has a higher motility. To measure FMD, we first drew regions of interest on the initial cell channel, away from the microchannels (as illustrated by red box, Fig. 3B). Each cell was registered into Imaris based on their mCherry signal, and the distance travelled by each of them were calculated. Therefore, FMD is fundamentally different from CMI, since FMD did not reflect the capability of cells squeezing through microchannels. FMDs were measured based on the migration distance of at least 100 cells in one chip unit, and the average of three chip units is reported.

CMI is defined here as the ratio between the number of nuclei (representing the cell) clearing the microchannels (exemplified by cells in hashtags, Fig. 3B), divided by the total number of nuclei located at the microchannels (exemplified by cells in asterisks, Fig. 3B). Higher CMI indicates those U87 cells which had a higher capability in infiltrating through tight spaces. The CMI of each group is calculated by averaging the counting made on at least three biological replicates.

To reveal the correlation between pSiNP uptake and cell migration, Cy5 fluorescence intensity in the cells, corresponding to the level of uptake, was measured as integrated density (IntDen). Cy5 intensity ratio, which equals to Cy5 intensity in cells cleared microchannels divided by Cy5 intensity in cells which did not, was reported. A low intensity ratio indicates that the migrated cells were less associated with pSiNP. Each intensity measurement is based on at least 50 randomly selected cells, and an average ratio over 3 chip units is reported for each group. In addition, the aspect ratio of FA, which reflects their stability^64,66^, was measured and reported. Images of vinculin immunofluorescence showed the morphology of FA were used. Using Fiji ImageJ, the length and the width of the FAs were measured. Aspect ratio (length divided by width) of the FA was reported. Measurement of aspect ratio for each group is based on at least 100 identifiable FA, an average aspect ratio is reported. A higher aspect ratio indicates the FA are more elongated. The aspect ratio is close to 1 when the FA are circular in shape.

### Cell ATP assay

Glo-luminescence cell viability assay kit (Promega, G7570) was used to measure the ATP content in cells treated by Tf@pSiNP, BSA@pSiNP, and Tf. Briefly, a starting density of 5,000 cells per well were first seeded onto 96-well white plates. The cells were maintained in medium for 1 or 2 d until confluence before treating the cells with the aforementioned groups. Control cells were those without any treatment, and each group was triplicated. After 8 h incubation, the cell viability was then determined using the assay kit following manufacturer provided protocol. The luminescence intensity of each group was then read using a microplate reader. The data were expressed as mean and standard deviation of 3 replicates.

### Scrape-migration assay

U87-mCherry cells in the logarithmic growth phase were collected and seeded into a 6-well plate at 37 °C in a humidified atmosphere of 5% CO_2_ for 24 h. When the cells reached above 80% confluence, a vertical scratch was introduced with a 10–200 μL micropipette tip. To keep the length and width of the scratch similar, the scratching angle was kept below 30°. To remove the detached cells, the plate was washed three time with PBS and then, immediately exposed to the previously prepared CO_2_ independent medium, with 10% serum medium containing the aforementioned groups or no treatment. The time-lapse images were captured every 30 min by laser scanning confocal microscope (Nikon Ti-E and A1R) for 14 h. The rate of cell migration was then calculated by measuring the percentage of scraped area covered over the period of the experiment. The rate was defined as (%) = (1 − scraped width/initial scratch width) × 100%

### Cell volume regulation measurement

U87-mCherry cells with the density of 10,000 cell/mL were seeded in glass-bottomed well plate and cultured for 8 h at 37 °C in 5% CO_2_ in a humidified incubator. Afterwards, the medium was replaced with DMEM containing Tf@pSiNP or no treatment and incubated for further 8 h. The well plate was placed on a laser scanning confocal microscope platform (SP8, Leica) equipped with a humidified incubator maintained at 37 °C and 5% CO_2_. A z-stack image was taken at the exact same point before and after adding hypertonic DMEM containing sucrose at a final concentration of 1 M sucrose.

The cytoplasmic volume of the cells, which was indicated by intracellular mCherry, was measured by Imaris software. Each measurement was based on at least 50 cells each chip, and an average over 3 chip units was generated for each group. Cell shrinkage ratio, which is defined by dividing the cell volume before and after hypertonic treatment, was reported. Larger cell shrinkage ratio indicates the cell volume reduced more under hypertonic treatment.

### Statistics

All experiments were triplicated, unless otherwise stated. Statistical analysis was conducted by using the Prism software. All data were presented as mean ± standard deviation (SD). The statistical difference between groups was determined by non-parametric Mann-Whitney test. The hypothesis was accepted at a 95% significant level (p < 0.05).

## Supplementary information

Supplementary information.

Supplementary information2.

Supplementary information3.
