# Supplementary material for: Transferrin-targeted porous silicon nanoparticles reduce glioblastoma cell migration across tight extracellular space
Source: Sci Rep. 2020 Feb 11;10:2320. doi: 10.1038/s41598-020-59146-5 (PMC7012928; doi:10.1038/s41598-020-59146-5)
Supplement: Supplementary file 1 — Supplementary information. [file 41598_2020_59146_MOESM1_ESM.pdf]

Supplementary Information

Transferrin-targeted porous silicon nanoparticles reduce glioblastoma cell migration across tight extracellular space

Sana Sheykhzadeh[a,b†], Meihua Luo[b,c†], Bo Peng[b,d,e], Jacinta White[d], Youssef Abdalla[a,b], Tweety Tang[b,f], Ermei Mäkilä[g], Nicolas H. Voelcker[b,c,d,e,h*], Wing Yin Tong[b,d,e*]

[a]Department of Pharmaceutical and Biological Chemistry, UCL School of Pharmacy, University College London, Brunswick Square, London, United Kingdom

[b]Drug Delivery, Disposition and Dynamics, Monash Institute of Pharmaceutics Science, Monash University, Parkville, Victoria, Australia

[c]Department of Biomedical Engineering, The Chinese University of Hong Kong, Shatin, New Territories, Hong Kong

[d]Commonwealth Scientific and Industrial Research Organization (CSIRO), Clayton, Victoria, Australia

[e]Melbourne Centre for Nanofabrication, Victorian Node of the Australian National Fabrication Facility, Clayton, Victoria, Australia

[f]Department of Biology & Chemistry, City University of Hong Kong, Hong Kong

[g]Industrial Physics Laboratory, Department of Physics and Astronomy, University of Turku, Turku, Finland

[h]Department of Materials Science and Engineering, Monash University, Clayton, Victoria, Australia

† Equally contributing first authors

* Correspondence: wingyin.tong@monash.edu, nicolas.voelcker@monash.edu

A

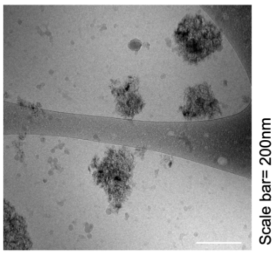

B

Nanoparticles	Size (nm)	PDI	Zeta(mv)
Tf@pSiNPs	182±0.8	0.1	-9.0±0.6
BSA@pSiNPs	172 ±4	0.1	-9.9±4.4

C

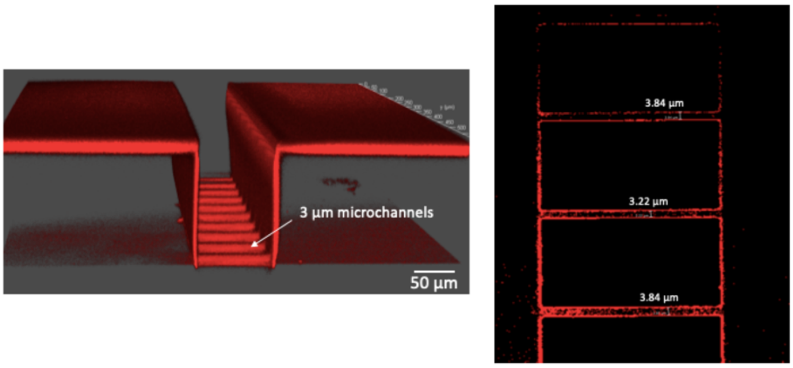

Supplementary Figure 1. A) Cryo-transmission electron microscopy image of BSA@pSiNP. B) Hydrodynamic size, PDI, and zeta potential of Tf@pSiNP and BSA@pSiNP measured by Zeta-sizer. C) Fluorescence microscopy images of Cy-5 labelled collagen I coated microchannel walls confirming the uniformity of the coating in the microchannels.

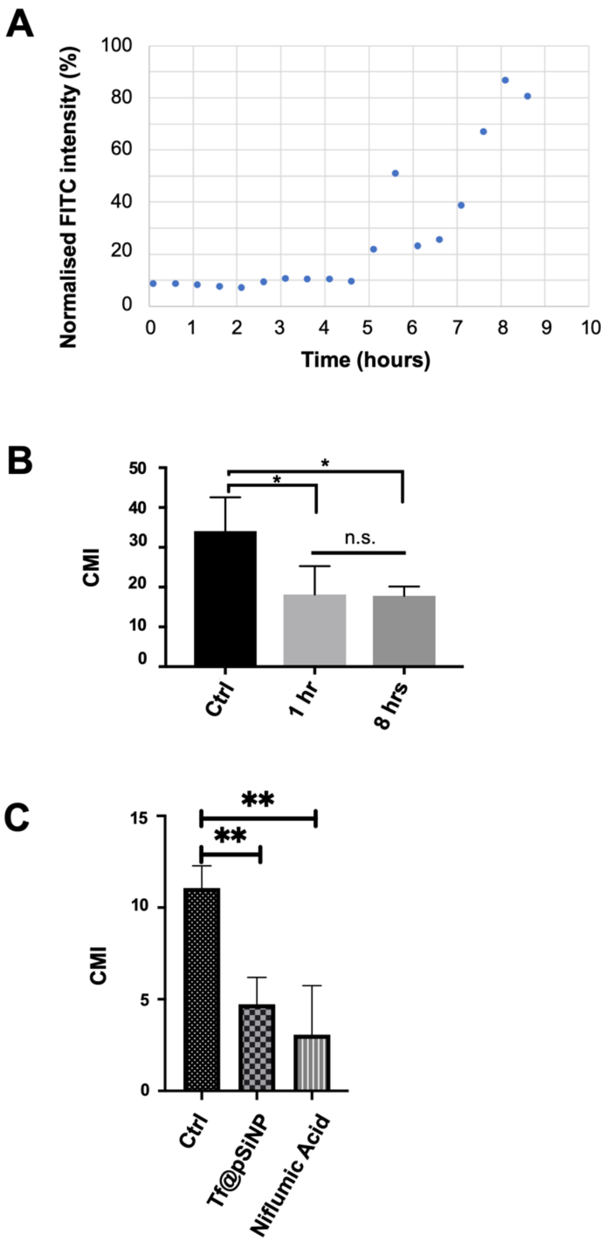

Supplementary Figure 2. A) Rate of mixing between initial cell channel and initial empty channel indicated by FITC-dextran concentration difference reported as normalised FITC intensity. An intensity of 100% indicates no concentration difference between the two channels. B) cell migration index (CMI) of U87-mCherry cells exposed to Tf@pSiNP for 1 h, 8 h, or left untreated (n= 3, error bar = ±1 SD). C) CMI of WK1 cells exposed to Tf@pSiNP and niflumic acid for 8 h using conventional Transwell model with pore size of 3 μm (n = 3, ** indicates $p<0.01$).

Supplementary Movie 1. Untreated U87-mCherry cells migrating though a section of microchannels, imaged at low magnification, over a period of 8 h. Red is cytoplasmic mCherry. Blue is nucleus.

Supplementary Movie 2. Talin expression of untreated migrating U87-mCherry cells across microchannels over a period of 18 min. Time interval between frames is 2 min. Green is talin. Red is cytoplasmic mCherry.
